# Production and Refinement of Omega-3 Rich Oils from Processing By-Products of Farmed Fish Species

**DOI:** 10.3390/foods8040125

**Published:** 2019-04-16

**Authors:** Vida Šimat, Jelena Vlahović, Barbara Soldo, Danijela Skroza, Ivica Ljubenkov, Ivana Generalić Mekinić

**Affiliations:** 1 Department of Marine Studies, University of Split, Ruđera Boškovića 37, HR-21000 Split, Croatia; 2 Sardina d.o.o., Ratac 1, HR-21410 Postira, Croatia; 3 Department of Chemistry, Faculty of Science, University of Split, Ruđera Boškovića 33, HR-21000 Split, Croatia; barbara@pmfst.hr (B.S.); ivica.ljubenkov4@gmail.com (I.L.); 4 Department of Food Technology and Biotechnology, Faculty of Chemistry and Technology, University of Split, Ruđera Boškovića 35, HR-21000 Split, Croatia; danci@ktf-split.hr (D.S.); gene@ktf-split.hr (I.G.M.)

**Keywords:** *Thunnus thynnus*, *Dicentrarhus labrax*, *Sparus aurata*, by-products, fish oil, chemical refining, fatty acid profile, volatile components

## Abstract

In this study, the effect of a four-stage chemical refining process (degumming, neutralization, bleaching, deodorization) on the quality parameters, fatty acid composition and volatile compounds of crude oils produced from processing by-products of farmed fish species (tuna, seabass and gilthead seabream) was evaluated. The quality of the oils was compared to commercially available cod liver oil on the basis of free fatty acid, peroxide value, *p*-anisidine, total oxidation (TOTOX), thiobarbituric acid reactive species (TBARS), oxidative stability at 80, 100 and 120 °C, tocopherol content, and volatile components, while the fatty acid profile and the proportion of polyunsaturated fatty acids (PUFAs) were used as an indicator of the nutritional values of fish oils. Quality parameters of the studied oils and oil oxidative stability were enhanced with refining and were within the limits recommended for fish oils without the loss of PUFAs. In tuna by-product refined oils, the proportion of PUFAs was over 40%, with 30% of eicosapentaenoic and docosahexaenoic fatty acids. The volatile compounds of the oils were quantified (in mg/kg) and major components were 2,4-heptadienal, pentadecane, 2,4-decadienal, 2,4-nonadienal and dodecane. The use of aquaculture by-products as an alternative source for fish oil production could contribute to a more sustainable and profitable aquaculture production, providing economic benefits for the producers and setting new standards for a fish by-product disposal strategy.

## 1. Introduction

The total aquaculture production of finfish is estimated at 54.1 million tonnes with a moderate annual growth rate of 5.8% with 37 world countries producing more farmed than wild-caught fish [1]. The Mediterranean and Black Sea areas have 62% of unsustainable fished stocks, particulary, the whitefish species. With the intent of making fisheries more productive and sustainable, mariculture (marine aquaculture) of seabass (*Dicentrarhus labrax*) and gilthead seabream (*Sparus aurata*) has grown exponentially, with these two species almost entirely farmed in the Mediterranean. The species with the highest commercial value in the Mediterranean is the Atlantic bluefin tuna (*Thunnus thynnus*) with capture-based mariculture (capture of fish from the wild and their rearing in sea cages for periods ranging between 3 months to 2 years) [2]. The growth of aquaculture also reflects on the generation of aquaculture by-products. In some countries such as Norway or Iceland, a large share of by-products is utilized by many different industries [3], in contrast to the Mediterranean region where this is not the practice. To our knowledge there is no available data for utilization of farmed tuna or seabass and gilthead seabream by-products for fish oil production or any other purpose that would benefit the producers economically and could serve as a reference for a fish by-product disposal strategy. 

The many benefits that marine lipids contribute to our overall health are well known and widely documented. High levels of nutritionally valuable long-chain polyunsaturated fatty acids (PUFAs), mainly eicosapentaenoic acid (EPA) and docosahexaenoic acid (DHA) along with their physicochemical properties, make fish lipids unique and a promising economically valuable product. Although the current situation with fish oil production is stable, future projections show that available wild marine resources are becoming increasingly limited while the demand for PUFA continues to rise. This not only suggests, but stresses the need for exploration and exploitation of alternative sources [4]. Rest raw materials of the fish processing industry, also known as by-products, vary within species and processing methods used, but generally include parts that remain after edible parts of the fish is removed: Heads, viscera, frames (bones with attached flesh), skins, fins, trimmings, blood and others not utilized for human consumption [5,6]. Rest raw materials should not be considered waste or less valuable than the main product [5]. The high nutritive value of fish by-products is related to the content of valuable minerals, vitamins, protein and lipid fractions thus they are used in wide range of purposes, from production of fish cakes, pies and nuggets, gelatin, sauces and other products for human consumption to production of biodiesel [7]. Bioactive components such as peptides, enzymes or collagens that are used by pharmaceutical, biomedical and biotechnological industries can also be extracted from fish by-products [1,6]. With the growing interest and scientific knowledge within the field of by-product utilization and properties of certain components such as protein and oil that can be extracted from fish by-products, the low-value products such as mince or fish meal have been replaced by products of higher value [5,8]. Different fish species have specific processing yields and consequently, generate different proportions of by-products. In addition, the quality and chemical composition of marine by-products vary between species, season and catching ground as well as post-capture handling and processing. Although not directly used for human consumption, ingredients from by-products are used as feed in terrestrial livestock industries and aquaculture farming and indirectly contribute to human food production. The possible limitations to by-product utilization are related to lower quality of the fish meal and fish oil in comparison to products obtained from whole fish and elevated enzymatic activity that makes them highly perishable. Several studies showed that oil from by-products can be successfully extracted and refined to remove some undesirable compounds (moisture, pigments, free fatty acids, phospholipids, minerals, off-flavours, etc.) which affect oil stability, overall quality and consumers’ acceptability, without the loss of PUFAs which enables its further application [9,10,11,12,13]. 

The aim of this research was to: (i) Produce and characterize crude oils from processing by-products obtained from farmed fish (tuna, seabass, and seabream); (ii) investigate the changes in fish oil characteristics during a four-stage refining process (degumming, neutralization, bleaching, deodorization) and differences in PUFAs composition between crude and refined oils; (iii) compare oils extracted from whole waste generated during tuna harvesting and tuna liver; (iv) report the characteristics of oil extracted from by-products obtained after filleting of farmed seabream and seabass; (v) compare the quality parameters and fatty acid composition of the obtained crude and refined oils with commercial cod liver oil in order to investigate the potential recycling of fish wastes for conversion into products of higher value. 

## 2. Materials and Methods

### 2.1. General

All the standards compounds, solvents and reagents used in the study were obtained from Sigma-Aldrich (St. Louis, MO, USA), Fluka (Neu-Ulm, Germany), Merck (Darmstadt, Germany) and Kemika (Zagreb, Croatia). Spectrophotometric measurements were performed on a SPECORD 200 Plus (Analytik Jena AG, Jena, Germany). The analyses of fatty acid methyl esters (FAMEs) and volatile compounds were carried out by gas chromatograph (GC, model 3900; Varian Inc., Lake Forest, CA, USA) with flame-ionization detection (FID). The oil oxidative stability was determined using Rancimat model 743 (Metrohm, Herisau, Switzerland). The α-tocopherol content was determined by high-performance liquid chromatography (HPLC, all components of Series 200; Perkin Elmer, Waltham, MA, USA).

### 2.2. Raw Materials and Fish Oil Extraction 

Farmed bluefin tuna (*Thunnus thynnus*) by-products were collected throughout December 2016 and January 2017 during harvesting and the evisceration process (done on board of fishing boat). By-products were separated into two groups: (i) Over 2000 kg of by-products (gills and gut content) were used for tuna by-product crude oil production; (ii) approximately 100 kg of tuna livers were collected (about 930 g each) and used for tuna liver crude oil production. During farming, tunas were fed with small pelagic fish such as sardines and herring and the average weight at harvesting was around 67 kg. Additionally, the farmed fish by-product crude oil was produced from approximately 1000 kg of heads, gills and gut content from seabream (*Sparus aurata*) and seabass (*Dicentrarhus labrax*) recovered from a local filleting plant. The fish were farmed at the same farm and fed with dry pellets (Efico plus, BioMar SAS, Nersac, France). Samples were 400–600 g at harvesting. Each oil was produced in two batches. Special attention was given to temperature regime during raw material manipulation. The crude oils were produced by grinding the raw materials in an industrial grinder (MG 250; Scansteel, Slagelse, Denmark), cooking at 95 °C for 12 min (cooking chamber model C2; Alfa Laval, Søborg, Denmark), and then pressed in a screw expeller and centrifuged at 4200 rpm using a decanter centrifuge type AC0303 CentryFish 1000 (Alfa Laval) which automatically separates dry matter (fishmeal), water and oil. With the interest of retaining the quality of crude oils, they were stored at <4 °C in dark bottles and refined within two days. Commercially available cod liver oil (oleum jecoris aselli; Kemig d.o.o., Zagreb, Croatia) was purchased from the local pharmacy and used as the control oil.

### 2.3. Oil Refinement Process

The refinement process, involving four major stages: Degumming (to separate phospholipids), neutralizing (to decrease acidity), bleaching (to remove coloured materials) and deodorization (to remove unwanted odour compounds), described by Chakraborty and Joseph [14] with slight modifications [15] was used. Extracted crude oil (500 g) was mixed with phosphoric acid (5 mL). The mixture was stirred in water bath heated to 70 °C for 20 min. After the cooling, samples were centrifuged (20 min, 4000 rpm) in order to remove the precipitated gum (degumming). Afterward, neutralization was done by slowly adding (drop by drop) NaOH solution (1 M) to the degummed oil samples in combination with constant stirring and heating at 65 °C for 20 min. The neutralization process was conducted until a pH of 7.0 was attained, and samples were then heated to 70 °C for 20 min, cooled and centrifuged for 15 min at 4000 rpm. The oil thus obtained was washed with deionized water (3-times with 10 mL) by agitation (500 rpm) and heating at 50 °C under vacuum. Neutralized oil samples were separated by centrifugation (10 min, 2500 rpm). The oil obtained after neutralization was bleached with adsorbents (4 g/100 g of oil) containing 1.13 g of activated carbon and 22.5 g of Fuller’s earth. The oil samples were stirred using a magnetic stirrer at 40 °C for 40 min under N_2_. After cooling, oil samples were again separated by centrifugation (30 min, 3500 rpm). Deodorization of the bleached oil samples was carried out by distillation under vacuum conditions. The mixture of the oil obtained after bleaching and deionized water (20 mL) was heated to 95–97 °C under the vacuum for one hour, with continual stirring. The refining process was repeated three times for each batch of oil.

### 2.4. Analytical Methodologies

American Oil Chemist’ Society [16] methodologies were followed for oils chemical characterization as follows: Acidic value (AV, Method no. Ca 5a-40) expressed as percent of oleic acid, peroxide value (PV, Method no. Cd 8-53) expressed as milliequivalents of O_2_/kg of oil, and anisidine values (*p*-AV, Method no. Cd 18-90) calculated as described in Chakraborty and Joseph [13]. The total oxidation (TOTOX) values were calculated as TOTOX = (2 × PV) + *p*-AV, while thiobarbituric acid reactive substances (TBARS) values were determined by the spectrophotometric assay described by Ke and Woyewoda [17]. The analyses were repeated five times.

The resistance of fish oil to auto-oxidation was determined at three different temperatures (80, 100 and 120 °C) by Rancimat method [18]. The fish oil (3 g) oxidative stability was tested at three different temperatures (80, 100 and 120 °C), while the airflow in all experiments was constant (20 L/h). The results were expressed as induction periods (IPs) which are the measure of oil stability or shelf-life, defined as the time in hours required to reach the end-point of oxidation.

### 2.5. Tocopherol Content

The tocopherol content was analyzed using HPLC system equipped with an autosampler, vacuum degasser, binary pump, fluorescent detector and the column oven on the Ultra Silica column (150 × 4.6 mm, 5 µm; Restek, Bellefonte, PA, USA) by a method previously described by Šimat et al. [15]. In short, an aliquot of 20 µL was obtained after dissolving 0.5 g of fish oil in 5 mL of hexane and injected into the chromatographic system. The following gradient elution program was applied using solvent A (hexane) and solvent B (isopropanol) at a flow rate of 0.9 mL/min: 3% B for the first 15 min, followed by an increase in solvent B to 80% through 5 min, after which the solvent ratio was maintained for 8 min. Over the next minute, the solvents returned to the initial conditions and this ratio of solvent was maintained for another 11 min to ensure column stabilization. The temperature of the column was held at 30 °C and detection observed by a fluorescent detector (excitation 290 nm and emission 330 nm). The compound was identified according to the retention time and quantified through the calibration curve of the standard. The analyses were performed in duplicate.

### 2.6. Fatty Acid Profile

The fatty acid methyl esters (FAMEs), prepared by dissolving oil samples (0.1 g) in heptane (2 mL) and by the addition of 2 M KOH in methanol (0.2 mL), were analyzed by gas chromatography with flame-ionisation detection (GC-FID) using capillary column RTX 2560 (100 m × 0.25 mm i.d., coating thickness 0.25 μm; Restek) by a method previously described in Šimat et al. [15]. In short, 1 µL of the heptane layer was injected into the chromatograph with a split ratio 1:100, with the temperature of injector et at 225 °C and of the detector at 240 °C. At a constant flow rate of 3 mL/min, helium was used as the carrier gas. The initial oven temperature was 140 °C, held for 5 min, raised to 240 °C at a rate of 4 °C/min and held at 240 °C for 20 min. FAMEs were identified by comparing to with standards (Supelco 37 Component FAME Mix; Sigma-Aldrich). The analyses were performed in duplicates and the results are expressed as percentages of methyl esters of individual fatty acids.

### 2.7. Analysis of Volatiles

The volatile compounds of oils were analyzed using headspace solid phase microextraction (HS-SPME) coupled with GC-FID. The samples were prepared using 2-cm long fibres of divinylbenzene-carboxen-polydimethylsiloxane (DVB-CAR-PDMS, thickness 50/30 µm) obtained from Supelco (Bellefonte, PA, USA). A total of 2 g of oil was placed into a 20 mL head space vial and equilibrated at 40 °C for 5 min, inserting the fibre into the headspace for adsorption at 40 °C for 30 min, then transferring the fibre to the injector port for desorption at 250 °C for 1 min. 

The quantitative analyses and separation of volatile compounds of major fish oil volatile components were performed by GC-FID and CP-WAX 57 CB column (50 m × 0.25 mm i.d., coating thickness 0.2 µm; Varian). At a constant flow rate of 2 mL/min, helium was used as the carrier gas. The GC oven temperature was programmed at an initial 40 °C for 4 min, raised to 190 °C at a rate of 5 °C/min and kept constant for 11 min. The temperature was then raised up to 200 °C applying the same rate of 5 °C/min. The detector temperature was maintained at 250 °C. The total analysis time for each sample was 67 min. The analyses were performed in duplicates.

The investigated compounds were identified by the retention time of the corresponding analytical standard, while the quantification was made by external calibration. As the applied method did not ensure adequate separation of 2,4-heptadienal and pentadecane, results for these two compounds are presented as their sum while their concentration was calculated using the calibration curve obtained for 2,4-heptadienal. GC Workstation Version 6.41 chromatographic software (Varian) was used for data collection and calculation [19].

### 2.8. Statistical Analysis

The obtained results are expressed as mean values ± standard deviation. The means of detected parameters were analyzed for significance by analysis of variance (one-way ANOVA) using Statgraphics Centurion (StatPoint Technologies Inc., Warrenton, VA, USA). Differences were considered to be significant at *p* < 0.05.

## 3. Results and Discussion

### 3.1. Chemical Characterization and Oxidative Stability of Fish Oils

The results of the chemical and compositional quality characterization of oils from tuna by-products, tuna liver, seabass and gilthead seabream by-products during four refining stages and their comparison to cod liver oil (control) are presented in Table 1. Despite the high temperature used during the oil production (cooking at 95 °C for 12 min) the temperature was found to have a poor influence on oil quality parameters. During preliminary studies we inspected quality parameters in oils extracted at different temperatures (from 65–95 °C) and compared it to Bligh and Dyer extracts [20] and found no significant difference in oxidative parameters [21]. It has been previously found that oil extraction temperature is weakly linked to the oxidative quality of produced oil, but strongly affected by omega-3 content of the raw material, providing confirmation of our finding [22]. To control the quality of fish oil properties, which is very labile to hydrolytic spoilage and oxidative deterioration, numerous standards with variable acceptable levels have been established [23]. The express lipolysis and oxidation of fish oils are the results of high autolytic activity and high content of PUFAs in fish tissues. It is expected that this process is even more susceptible for fish by-products. For this reason, fish oils usually have high free fatty acid (FFA) content. In this study, the results of FFA values of crude oils were low confirming that short cooking periods during the oil extraction, even at higher temperatures, did not cause significant hydrolysis. Only in tuna liver oil the FFA values were over 3. The refinement process ensured an additional decrease of FFA by 3.7, 32 and 47% in tuna by-product, tuna liver and seabass/seabream oil, respectively. Among the three studied oils, seabass and gilthead seabream by-product oil had the lowest FFA values, even lower than the control. The allowable limit of FFAs value for crude fish oil is in the range of 1–7% of oleic acid, usually 2–5% [24], but the general recommendation is that FFA values of edible oils should be ≤3.0%. This is important since FFAs have an impact on the oil organoleptic properties as well as oil compositional quality [25], can act as pro-oxidants which initiate the oxidation mechanism [26] and high FFA values are problematic during omega-3 extraction and biodiesel production [27]. 

Primary oxidation of oil used to monitor hydroperoxides formation is determined by PV and should be ≤5 meq O_2_/kg for fish oils intended for human consumption [28,29]. Despite the increase of the PVs for studied oils after degumming step, final values obtained for the refined oils were below the limit of 5 meq O_2_/kg (Table 1) which opens the possibility of using these oils for human consumption.

The influences of the refining steps on oil oxidation status and products expressed as *p*-AV, TOTOX and TBARS are presented in Table 1. Among crude oils, *p*-AV and TOTOX values for tuna by-product oil were the highest, followed by those for tuna liver oil and seabass and gilthead seabream by-product oil. The processing steps of degumming, neutralizing and bleaching caused reduction of *p*-AV and TOTOX while these parameters increased after the deodorization step. The highest *p*-AV value was detected for refined tuna by-product oil (19.5), while TOTOX values of refined tuna liver and seabass and gilthead seabream by-product oils were higher than those obtained for crude oils, 27.7 and 24.7, respectively. This suggests that used adsorbents (activated carbon and Fuller’s earth) have the capacity to adsorb primary and secondary oxidation compounds [14]. The allowable limit of *p*-AV for acceptability of fish oil for human consumption is ≤20 [30]. 

Although the *p*-AV obtained for oils in this study were under this limit, they were significantly higher than the control, thus additionally influencing the higher TOTOX value. The TOTOX is a parameter used to determine the presence of compounds generated by degradation of PUFAs under pro-oxidant conditions including high temperatures, oxygen, metal compounds and light, and the TOTOX value ≤ 26 under is found to be allowable for fish oil [31]. Therefore, in association to the above mentioned PV and *p*-AV values, the TOTOX values of studied by-product oils were above the mentioned limit in crude and refined oils from tuna by-products and just under the limit in seabass and seabream by-product oil (Table 1). The *p*-AV value and TOTOX obtained for crude sardine oil were found to be 16 and 40, respectively [14]. In that study, authors applied the same 4-stage refining process which was more effective for sardine oil and reduced *p*-AV value and TOTOX values to 10 and 19, respectively. On the other hand, as reported in Table 1, TBARS assay detected low accumulation of secondary oxidation products. TBARS detects lipid oxidation when thiobarbituric acid and oxidation products from unsaturated FAs react involving several secondary oxidation products, however, the TBARS of by-product oils was lower that the control oil.

**Figure 1:**
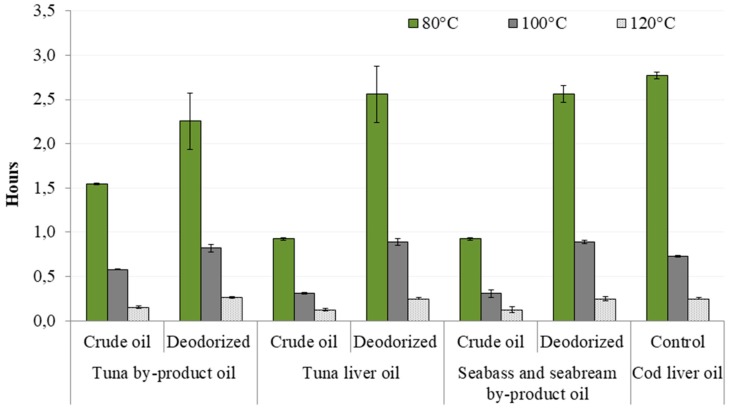
The oxidative stability (*n* = 18) of crude and deodorized (refined) oils from tuna by-products, tuna liver, seabass and gilthead seabream by-products, and cod liver oil (control).

**Figure 2:**
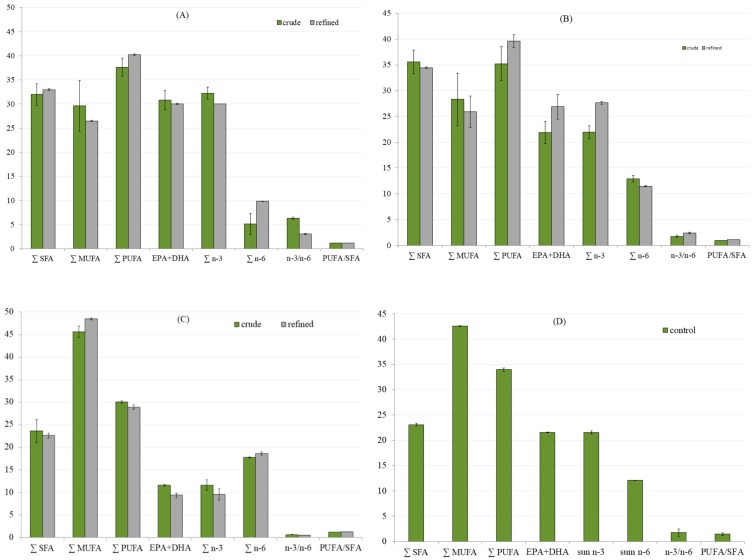
The sum of monounsaturated (∑MUFA) and polyunsaturated (∑PUFA) fatty acids, the sum of eicosapentaenoic and docosahexaenoic fatty acids (EPA + DHA), the sum of *n-3* and *n-6* content plus ratios between *n-6/n-3* and polyunsaturated fatty acids/saturated fatty acids (PUFA/SFA) in crude and refined oils from: (**A**) tuna by-products, (**B**) tuna liver, (**C**) seabass and gilthead seabream by-products and (**D**) cod liver oil (control).

**Figure 3:**
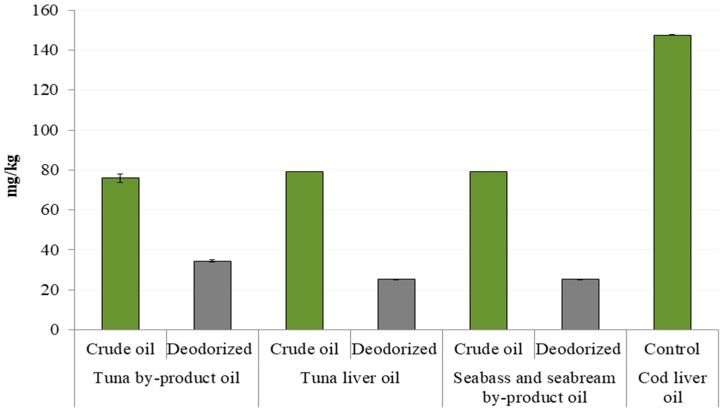
The tocopherol content (*n* = 12) in crude and deodorized (refined) oils from tuna by-products, tuna liver, seabass and gilthead seabream by-products and cod liver oil (control).

The Rancimat method, an accelerated method that employs high temperatures and air-flow supply to estimate the oxidative stability and shelf life of oil-containing products in a relatively short time, was used to measure the oxidative stability of oils (Figure 1). According to the presented results it can be seen that tuna by-product oil was the most stabile sample among crude oils with IP of 1.54 h at 80 °C, 0.58 h at 100 °C and 0.15 h at 120 °C, while lower values were obtained for the other two oils. The refinement process prolonged the oil oxidative stability in all cases, but unlike for crude oils, refined tuna liver oil and seabass and seabream by-product oil showed higher IP values than tuna by-product oil (prolongation of 36% at 80 °C, 35% at 100 °C and 52 % at 120 °C). The oil resistance to the lipid oxidation is a result of differences between fatty acid profiles of investigated oils. From the Table 2 and Figure 2 it can be seen that crude tuna by product oil contain the highest content of PUFAs (∑ of 37.65%), as well as EPA + DHA (∑ of 30.85%). Furthermore, the *n-3/n-6* ratio in crude tuna waste oil was more than 3.5-fold higher than in crude tuna liver oil, and more than 9.5-fold higher than in crude seabass and seabream by-product oil. The refinement process reduced this parameter by half in tuna by-product oil, slight reduction was obtained in seabass and seabream by-product oil, while a higher value was detected in refined tuna liver oil. Although the referent cod liver oil showed the highest stability at 80 °C, at higher temperatures its stability was lower that of the investigated refined oils. As can be seen in Figure 3, significantly higher content of tocopherol in cod liver oil has been detected. This compound has been added during the production of the commercial oil sample in order to improve it stability, but it has been established that tocopherol degrades at higher temperatures [32] which is probably caused lower IP of the cod liver at 100 and 120 °C.

### 3.2. Fatty Acid Profile of Oils

The results of the fatty acid profile of the crude oils and their changes after oil refinement are presented in Table 2 and in Figure 2A–D. The most dominant SFA contributing approximately 52–55% of total SFA, in all investigate oils was palmitic FA (C16:0). Its content was significantly higher in tuna oils in comparison to seabream/seabass oil and the control. Palmitic FA occurs naturally in fish, being a source of metabolic energy for their growth. The oleic acid (C18:1*n-9 cis*) was the major compound among MUFAs and in the studied oils amounted to approximately 14% in tuna oils, to 40% in seabass and gilthead seabream by-product refined oil. Among PUFAs, high concentrations of EPA (C20:5*n-3*) and even higher those of DHA (C22:6*n-3*) were found in all studied oils. The relative contents of EPA and DHA were expected to increase during the refining process [14,33], but in this study the minimal increase was observed after refinement only for DHA in tuna liver oil. However, in both crude and refined tuna by-product oils amounts of EPA and DHA were found to be extremely high. In tuna oils and the control oil, especially in tuna liver oil dihomo-γ-linolenic acid (20:3*n-6*), was found in higher amounts (7.32-9.89%), while in seabream/seabass oil its precursor, linoleic acid (C18:2*n-6*) was found significantly higher compared to other oils (17.32%). Erucic acid (22:1*n-9*) was found in small amounts in studied oils, with the highest content (1%) found in the control oil.

The crude oils from tuna by-products and liver contained significantly higher total amounts of saturated fatty acids (32.7 and 35.6%, Figure 2) in comparison to seabass and gilthead seabream by-product oil (23.6%). The SFA profile of the oils did not statistically change with the refining process and tuna oils had significantly higher SFA content than the control. The MUFAs content was the highest in seabass and gilthead seabream by-product oil (46%) and did not change after refining. Tuna by-product oil had slightly higher MUFA content then tuna liver oil. The PUFAs were significantly higher in tuna by-product and tuna liver crude oils (37.7 and 35.2%) and they increased to 40.2 and 39.6% in refined oils, respectively. The content of PUFA*n-3*, in the studied oils, ranged from 11.6–32.3% and was higher than that of PUFA*n-6* for 8.9-18.7% (Figure 2). A high percentage of PUFA indicates good nutritional values of the studied fish oils. The PUFA/SFA ratio of 0.4-0.5 is considered beneficial for human health [34], and it was significantly higher in the crude oils, while in refined oils it ranged from 1.1 to 1.2, significantly lower than in the control oil (Figure 2). In general, it is accepted that compared to wild caught fish, modern aquaculture products have lower *n-3* FAs, and higher levels of terrestrial plant-originating C18:2*n-6* as a result of feed composition [35]. Very high intake of *n-6* was recognized as undesirable and it reduces the nutritional quality of fish oil. The sum of n-6 was 18-19% higher in seabass and gilthead seabream by-product oils and along with the lowest sum of *n-3* and EPA+DHA. 

In tuna by-product oils the refining process resulted with a decrease share of MUFAs and the increase of PUFAs. This, as well as the content of antioxidant compounds such as tocopherol (Figure 3), can contribute to the oxidative stability of oils. Crude oils from farmed fish by-products had high tocopherol content which was significantly reduced by refining process in all oils (31–45%). At the same time, oxidative stability was prolonged by the refining process thus the role of tocopherol in oxidative stability appears to be smaller than the removal impurities which act as pro-oxidants [36]. This is confirmed on the control oil which had over 140 mg of tocopherol per kilogram (data from declaration sheet indicate a content of 1190 International Units of vitamin A per gram) however this content did not enhance its oxidative stability at elevated temperatures. The tocopherol content of oils from farmed fish species is higher in comparison to oils from wild fish and their by-products. For example, sardine by-product crude oil has approximately 30 mg tocopherol /kg [15]. It has been suggested that dietary elements of fish feed, such as vitamin E, do not influence significantly the amount of total lipids, phospholipids, polyunsaturated and general muscle fatty acid composition but protect from peroxide formation and phospholipid hydrolysis [35]. 

### 3.3. Volatile Profile of Oils

The composition and relative contents of volatile compounds of crude, bleached and deodorized oils from tuna by-products, tuna liver, seabass and gilthead seabream by-products and cod liver oil (control) are presented in Table 3. Sixteen volatile components were identified (one ester, six aldehydes, five alcohols and three hydrocarbons). In tuna by-product oil the most dominant was the sum of 2,4-heptadienal (from *n-3* fatty acids) and pentadecane, followed by (E,E)-2,4-decadienal, dodecane and 4-methylpenten-2-ol. The applied GC-FID method did not ensure adequate separation of 2,4-heptadienal and pentadecane, and their concentration was calculated using the calibration curve obtained for 2,4-heptadienal since it was considered more important in fish oil, however the exact amount of the 2,4-heptadienal in the total sum is unknown. Taking into account that other volatile compounds such as (E,Z)-2,6-nonadienal and (E,E)-2,4-decadienal (secondary lipid oxidation products) were found in low concentrations, low TBARs values (Table 1) and previous reports that suggest pentadecane as dominant component in fish oil samples [37,38], we can assume that pentadecane is the dominant compound in this mixture. Similar was observed for tuna liver oil with exception to high levels of 1-penten-3-ol. Among unsaturated alcohols responsible for the fishy odour of the oil, one of the most important components is 1-pentene-3-ol [39]. The content of this compound was significantly reduced in all studied oils and in refined oils, with the findings ranging from 0.01–1.03 mg/kg. 

In seabass and gilthead seabream by-products oil aldehydes, (E,E)-2,4-decadienal and 2,4-nonadienal, were found to be higher than in tuna by-product oils and levels of 2,4-heptadienal+pentadecane were significantly lower. The amount of 2,4-decadienal (from the *n-6* fatty acids) increased after the distillation, especially in seabass and seabream oil. In comparison to studied oils, the control oil was characterized with high levels of tetradecene. The values of two fatty aldehydes, 2,4-heptadienal and 2,4-decadienal are of great importance due to their contribution to the characteristic unpleasant odour of the oil [14,39]. The composition and proportion of volatile compounds changed significantly during the refining process and only in tuna by-product oil the total sum of volatile compounds was reduced during refining. The aldehydes are known as essential indicators of the oxidation processes and are also responsible for oil fishy odour, same for ketones which usually have very low thresholds and are derived from autoxidation of PUFAs via hydroperoxides or lipid oxidative degradation. In order to remove undesirable favor components, such as oxidation products (aldehydes and ketone, residual free fatty acids, etc.) the deodorization step is generally carried out by conventional steam distillation at temperatures below 200 °C. The suggested procedure does not affect all volatile components equally. The effectiveness of this process is influenced by the applied pressure and volatility of components at high temperature. In this stage, it is also important to inhibit the degradation of the essential components by cyclization and polymerization of long chain PUFAs [26].

Chakraborty and Joseph [14] reported that sardine fish oil distillate obtained after 60 min of steam distillation under vacuum contained two prominent aldehydes, namely, 2,4-heptadienal and 2,4-decadienal. The concentrations of these compounds were also high in our study suggesting that improvement of the distillation method is necessary. 

The aroma components of fish have been widely studied, however volatile compound profiles in crude fish by-products oils and in oils undergoing the refining process have rarely been reported. In general, volatile compounds of fish oils are only identified [14,33,40], without dealing with the quantification of those components (concentrations reported in mg/kg instead of in % of peak area). The volatile components in fish oils are usually a result of microbiological spoilage or oxidation processes of lipids, amino acids and proteins and the knowledge of their chemical characterization and changes during treatments is useful for feed and aquatic industry as it opens new usage possibilities of different processing by-products [14,33,41]. 

The changes of volatile profiles of fish oil, from tuna and anchovy by-products in chemical refining process have been previously reported by Song et al. [33]. Authors identified 63 volatile compounds, with hexanal, nonanal, undecanal, 2-nonanone, and 2-undecanone being the key volatile components of the fish oils. The study demonstrated that compounds which are most responsible for the unfavorable odour of the fish oil could be effectively removed by the refining process which directly enhances the oil quality. Oliveira et al. [42] studied the effects of chemical refining and deodorization on fatty acid profiles and sensory characteristics of the tuna (*Thunnus albacares*) by-product oil obtained by enzymatic hydrolysis. The oil was extracted from the heads and was found rich in PUFAs. In comparison to this study, authors found higher content of MUFAs in refined oil (36.78%) and lower content of PUFA (33.18%) and recommended deodorization conditions at 160 °C for 1 h and 200 °C for 1 h for PUFA rich oils. 

## 4. Conclusions

The chemical and compositional quality of the characterization of oils from tuna by-products, tuna liver, seabass, and gilthead seabream by-products during the four stage refining process suggests that by-products are suitable and valuable raw material for PUFA-rich oil production. Crude oils showed good characteristics; and the four-stage refining process was effective in reducing oil impurities resulting in lower FFA, PV, *p*-AV, TOTOX, and TBARS values of studied oils. Refined oils also showed better oxidative stability. A beneficial compound, α-tocopherol, which might improve the oil quality was lowered by the described refining process in all oils. Volatile compounds responsible for the sensory profile of the oils were formed and removed from the oils during different refining steps without clear relation to FFA and TBARS values. In order to maintain high levels of long-chain fatty acids, such as EPA and DHA, the described refining procedure was effective, but for effective removal of volatile components from fish oil, improvement of the deodorization step is needed possibly at temperatures higher than 100 °C for a short period of time. During the search of an alternative source of raw material for fish oil production, the results of this paper have been found to contribute to a more efficient utilization of natural marine resources, clearly indicating that by-products may offer a solution that will improve waste management, an ecological aspect of fish processing, thereby adding value to “waste”.

## Figures and Tables

**Table 1:** Chemical and quality parameters of crude oils from tuna by-products, tuna liver, seabass and gilthead seabream by-products at different stages of the refining process, and cod liver oil (control).

Oil Samples	Refining Phase	Measured Parameter				
		FFA (% Oleic Acid)	PV (meq O_2_/kg)	*p*-AV	TOTOX	TBARS (μM/g)
Tuna by-product oil	*Crude oil*	2.94 ± 0.12 ^a^	2.31 ± 0.73 ^a^	25.2 ± 0.08 ^a^	29.7 ± 0.20 ^a^	0.94 ± 0.33 ^a^
	*Degummed*	3.51 ± 0.03 ^b^	3.93 ± 0.18 ^b^	19.2 ± 1.25 ^b^	27.1 ± 0.79 ^b^	1.02 ± 0.14 ^a^
	*Neutralized*	3.13 ± 0.10 ^b^	3.46 ± 0.39 ^b^	16.9 ± 1.23 ^c^	23.8 ± 1.06 ^c^	2.02 ± 0.45 ^b^
	*Bleached*	2.93 ± 0.04 ^a^	2.88 ± 0.11 ^ab^	14.4 ± 0.90 ^d^	20.2 ± 1.34 ^d^	1.88 ± 0.37 ^b^
	*Deodorized*	2.83 ± 0.01^a^	3.78 ± 0.22 ^b^*	19.5 ± 0.94 ^b^	27.1 ± 2.15 ^b^	1.14 ± 0.11 ^a^
Tuna liver oil	*Crude oil*	3.13 ± 0.00 ^a^	2.68 ± 0.86 ^a^	20.9 ± 1.34 ^a^	26.0 ± 0.14 ^a^	2.88 ± 0.73 ^a^
	*Degummed*	2.52 ± 0.05 ^b^	3.01 ± 0.05 ^ab^	19.1 ± 2.47 ^b^	25.2 ± 1.17 ^a^	1.35 ± 0.02 ^b^
	*Neutralized*	2.65 ± 0.11 ^c^	2.89 ± 0.22 ^ab^	17.0 ± 4.78 ^c^	22.7 ±1.22 ^b^	1.76 ± 0.03 ^b^
	*Bleached*	2.17 ± 0.03 ^b^	2.85 ± 0.53 ^ab^	14.0 ± 0.65 ^d^	19.7 ± 0.43 ^c^	1.92 ± 0.35 ^b^
	*Deodorized*	2.12 ± 0.16 ^b^	4.25 ± 0.28 ^c^	19.2 ± 2.22 ^b^	27.7 ± 2.22 ^a^	1.97 ± 0.53 ^b^
Seabass and gilthead seabream by-product oil	*Crude oil*	2.41 ± 0.47 ^a^	4.63 ± 1.07 ^a^	18.1 ± 0.19 ^ab^	19.3 ± 2.45 ^a^	0.53 ± 0.05 ^a^
	*Degummed*	1.66 ± 0.05 ^b^	2.70 ± 0.18 ^b^	15.7 ± 1.12 ^c^	20.2 ± 0.12 ^a^	3.46 ± 2.08 ^b^
	*Neutralized*	1.43 ± 0.18 ^b^	3.13 ± 0.31 ^b^	12.7 ± 0.65 ^a^	18.9 ± 0.26 ^a^	2.24 ± 1.05 ^bc^
	*Bleached*	1.40 ± 0.01 ^b^	3.44 ± 0.25 ^ab^	11.4 ± 0.19 ^b^	18.4 ± 0.52 ^a^	1.14 ± 0.10 ^ac^
	*Deodorized*	1.28 ± 0.15 ^b^	4.20 ± 0.15 ^ab^	16.5 ± 0.39 ^c^	24.7 ± 0.32 ^b^	1.42 ± 0.53 ^ac^*
Cod liver oil	*Control*	2.15 ± 0.08	3.85 ± 0.44	13.3 ± 1.11	21.0 ± 0.18	1.63 ± 0.07

*n* = 30; Different superscript letters (a–d) in the same column denote statistically significant difference (*p* < 0.05); * Measured parameters in deodorized oils marked with * do not differ statistically (*p* < 0.05) from the control sample.

**Table 2:** The changes in fatty acid profile (%) of crude and refined oils from tuna by-products, tuna liver, seabass and gilthead seabream by-products and comparison to cod liver oil (control).

Fatty Acid	Tuna By-Product Oil		Tuna Liver Oil		Seabass and Seabream By-Product Oil		Cod Liver Oil
	*Crude Oil*	*Refined*	*Crude Oil*	*Refined*	*Crude Oil*	*Refined*	*Control*
**C12:0**	n.d. ^1^	0.06 ± 0.00	0.07 ± 0.00	0.06 ± 0.01	0.02 ± 0.01	0.03 ± 0.00	0.02 ± 0.02
**C13:0**	n.d.	0.06 ± 0.00	0.07 ± 0.00	0.07 ± 0.02	n.d.	0.02 ± 0.00	0.01 ± 0.01
**C14:0**	6.08 ± 0.98	6.05 ± 0.00	6.60 ± 0.49	6.50 ± 0.25	2.31 ± 0.02	2.57 ± 0.14	4.64 ± 0.02
**C14:1**	0.46 ± 0.65	0.04 ± 0.06	0.91 ± 0.06	n.d.	0.36 ± 0.00	0.40 ± 0.00	0.39 ± 0.03
**C15:0**	0.37 ± 0.52	0.82 ± 0.01	0.07 ± 0.00	0.88 ± 0.02	0.04 ± 0.01	0.05 ± 0.00	0.12 ± 0.01
**C15:1**	n.d.	0.01 ± 0.01	0.01 ± 0.00	n.d.	n.d.	n.d.	n.d.
**C16:0**	18.20 ± 2.12	18.09 ± 0.23	19.85 ± 1.54	18.97 ± 0.82	12.70 ± 0.02	13.87 ± 0.23	12.16 ± 0.18
**C16:1**	6.14 ± 0.74	6.09 ± 0.06	5.61 ± 0.43	5.40 ± 0.22	3.55 ± 0.09	3.88 ± 0.17	8.81 ± 0.01
**C17:0**	0.67 ± 0.07	0.65 ± 0.00	0.75 ± 0.06	0.71 ± 0.03	0.32 ± 0.02	0.34 ± 0.01	0.19 ± 0.02
**C17:1**	0.46 ± 0.10	0.44 ± 0.01	0.40 ± 0.04	0.35 ± 0.02	0.27 ± 0.01	0.29 ± 0.01	0.35 ± 0.02
**C18:0**	4.28 ± 0.36	4.23 ± 0.01	4.97 ± 0.36	4.66 ± 0.08	2.63 ± 0.01	0.17 ± 0.00	3.21 ± 0.34
**C18:1n-9t**	2.64 ± 0.41	0.16 ± 0.00	0.17 ± 0.02	0.16 ± 0.01	0.12 ± 0.00	0.15 ± 0.00	0.14 ± 0.00
**C18:1n-9c**	13.92 ± 1.14	13.73 ± 0.18	13.42 ± 1.09	13.05 ± 0.09	37.97 ± 0.08	40.52 ± 0.72	18.73 ± 0.01
**C18:2n-6t**	0.06 ± 0.08	0.05 ± 0.03	0.08 ± 0.09	0.13 ± 0.01	0.04 ± 0.00	0.04 ± 0.00	0.08 ± 0.00
**C18:2n-6c**	1.04 ± 1.47	2.18 ± 0.01	2.28 ± 0.18	2.13 ± 0.02	16.35 ± 0.02	17.32 ± 0.26	1.89 ± 0.02
**C20:0**	0.42 ± 0.03	0.42 ± 0.00	0.47 ± 0.04	0.43 ± 0.01	0.51 ± 0.00	0.39 ± 0.01	0.29 ± 0.01
**C18:3n-6**	0.39 ± 0.55	0.13 ± 0.00	0.12 ± 0.01	0.11 ± 0.02	0.19 ± 0.00	0.19 ± 0.00	0.15 ± 0.00
**C20:1**	5.07 ± 0.20	5.08 ± 0.02	6.50 ± 0.56	5.84 ± 0.04	2.47 ± 0.00	2.50 ± 0.05	12.67 ± 0.04
**C18:3n-3**	1.41 ± 0.12	n.d.	n.d.	0.65 ± 0.06	0.02 ± 0.02	0.18 ± 0.00	n.d.
**C21:0**	n.d.	1.44 ± 0.04	1.48 ± 0.13	0.67 ± 0.08	4.28 ± 0.01	4.46 ± 0.05	0.90 ± 0.02
**C20:2**	0.28 ± 0.01	0.27 ± 0.01	0.31 ± 0.03	0.11 ± 0.01	0.59 ± 0.01	0.58 ± 0.02	0.30 ± 0.02
**C22:0**	0.08 ± 0.11	0.15 ± 0.01	0.18 ± 0.02	0.17 ± 0.02	0.19 ± 0.02	0.17 ± 0.01	0.14 ± 0.00
**C20:3n-6**	3.54 ± 5.01	7.32 ± 0.02	10.25 ± 0.92	9.04 ± 0.17	1.04 ± 0.00	0.97 ± 0.01	9.89 ± 0.02
**C22:1n-9**	0.32 ± 0.01	0.30 ± 0.00	0.39 ± 0.03	0.37 ± 0.01	0.46 ± 0.03	0.43 ± 0.01	1.00 ± 0.02
**C20:3n-3**	n.d.	0.02 ± 0.00	0.05 ± 0.00	0.11 ± 0.11	0.03 ± 0.00	0.02 ± 0.00	n.d.
**C20:4n-6**	0.19 ± 0.13	0.18 ± 0.00	0.19 ± 0.01	0.09 ± 0.14	0.16 ± 0.00	0.16 ± 0.00	0.16 ± 0.01
**C23:0**	1.02 ± 0.08	0.97 ± 0.00	1.01 ± 0.07	1.92 ± 0.01	0.51 ± 0.01	0.48 ± 0.01	0.58 ± 0.01
**C22:2**	n.d.	0.03 ± 0.00	0.05 ± 0.01	0.36 ± 0.07	0.05 ± 0.01	0.05 ± 0.00	0.02 ± 0.01
**C24:0**	0.87 ± 0.04	0.02 ± 0.00	0.07 ± 0.01	0.40 ± 0.02	0.07 ± 0.00	0.05 ± 0.01	0.82 ± 0.04
**C20:5n-3**	9.56 ± 0.76	9.29 ± 0.04	8.81 ± 0.68	8.21 ± 0.03	3.33 ± 0.03	3.04 ± 0.00	9.58 ± 0.06
**C24:1**	0.60 ± 0.02	0.65 ± 0.00	0.90 ± 0.09	0.77 ± 0.14	0.45 ± 0.01	0.34 ± 0.04	0.48 ± 0.00
**C22:6n-3**	21.29 ± 1.09	20.75 ± 0.19	13.07 ± 7.00	18.66 ± 1.21	8.26 ± 0.24	6.37 ± 0.47	11.9 ± 0.26

^1^ n.d.—not detected; *n* = 12.

**Table 3:** The concentration of volatiles (mg/kg) in crude oils from tuna by-products, tuna liver, seabass and gilthead seabream by-products at different stages of the refining process, and cod liver oil (control).

	Volatile Compound	Tuna By-Product Oil			Tuna Liver Oil			Seabass and Seabream By-Product Oil			Cod Liver Oil
		*Crude*	*Bleached*	*Deodorized*	*Crude*	*Bleached*	*Deodorized*	*Crude*	*Bleached*	*Deodorized*	*Control*
**Esters**	Ethyl acetate	1.51 ± 0.14 ^a^	1.45 ± 0.19 ^a^	1.00 ± 0.02 ^b^*	0.93 ± 0.28 ^a^	1.03 ± 0.29 ^a^	1.19 ± 0.01 ^a^*	0.50 ± 0.03 ^a^	1.69 ± 0.25 ^b^	0.99 ± 0.07 ^c^*	0.73 ± 0.07
**Aldehydes**	Pentanal	1.12 ± 0.14 ^a^	1.55 ± 0.06 ^a^	1.94 ± 0.32 ^a^*	0.95 ± 0.20 ^ab^	0.81 ± 0.05 ^ab^	0.54 ± 0.01 ^bc^	2.09 ± 0.05 ^a^	1.53 ± 0.11 ^b^	0.55 ± 0.04 ^c^	1.22 ± 0.11
	E-2-hexenal	0.08 ± 0.01 ^a^	0.05 ± 0.01 ^a^	0.05 ± 0.02 ^a^	0.04 ± 0.01 ^a^	0.04 ± 0.01 ^a^	0.02 ± 0.0 ^a^	0.01 ± 0.0 ^a^	0.01 ± 0.0 ^a^	0.02 ± 0.0 ^a^	0.51 ± 0.04
	Octanal	0.29 ± 0.25 ^a^	0.53 ± 0.0 ^a^	0.39 ± 0.24 ^a^*	1.15 ± 0.08 ^a^	0.41 ± 0.20 ^a^	0.45 ± 0.10 ^a^*	0.34 ± 0.13 ^ab^	0.31 ± 0.02 ^ab^	0.15 ± 0.09 ^a^*	0.14 ± 0.02
	(E,Z)-2,6-nonadienal	0.98 ± 0.21 ^a^	1.07 ± 0.14 ^a^	0.85 ± 0.13 ^a^	0.91 ± 0.01 ^a^	1.20 ± 0.16 ^a^	1.08 ± 0.08 ^a^	0.40 ± 0.06 ^a^	0.62 ± 0.09 ^b^	0.65 ± 0.03 ^b^	0.14 ± 0.0
	(E,E)-2,4-decadienal	4.59 ± 0.28 ^a^	6.06 ± 0.57 ^b^	4.94 ± 0.35 ^ab^	6.28 ± 1.19 ^a^	6.25 ± 0.70 ^a^	6.76 ± 0.42 ^a^*	11.5 ± 0.46 ^a^	10.3 ± 0.18 ^a^	14.1 ± 0.54 ^b^	6.80 ± 0.21
	2,4-nonadienal	0.67 ± 0.04 ^a^	1.03 ± 0.14 ^a^	1.02 ± 0.13 ^a^	2.09 ± 0.16 ^a^	0.93 ± 0.13 ^b^	0.96 ± 0.13 ^b^	6.53 ± 0.58 ^a^	6.59 ± 0.49 ^a^	4.77 ± 0.02 ^b^	0.26 ± 0.05
**Alcohols**	1-penten-3-ol	0.08 ± 0.0 ^a^	0.05 ± 0.01 ^ab^	0.01 ± 0.01 ^b^*	11.17 ± 0.20 ^a^	2.29 ± 1.31 ^b^	1.03 ± 0.0 ^b^	0.65 ± 0.01 ^a^	0.37 ± 0.02 ^b^	0.07 ± 0.0 ^c^	0.02 ± 0.0
	4-methylpenten-2-ol	2.66 ± 0.07 ^a^	1.42 ± 0.17 ^b^	2.15 ± 0.57 ^a^	1.14 ± 0.03 ^a^	1.08 ± 0.57 ^a^	1.46 ± 0.0 ^a^*	0.57 ± 0.03 ^a^	1.55 ± 0.10 ^b^	1.91 ± 0.10 ^c^	1.14 ± 0.03
	Hexanol	0.08 ± 0.01 ^a^	0.03 ± 0.01 ^b^	0.05 ± 0.03 ^b^*	0.06 ± 0.0 ^a^	0.03 ± 0.01 ^a^	0.04 ± 0.0 ^a^*	0.06 ± 0.0 ^a^	0.02 ± 0.0 ^b^	0.03 ± 0.0 ^c^	0.06 ± 0.01
	E-2-hexen-1-ol	0.08 ± 0.0 ^a^	0.06 ± 0.04 ^ab^	0.11 ± 0.03 ^a^	0.01 ± 0.01 ^a^	0.13 ± 0.09 ^a^	0.23 ± 0.02 ^a^	0.04 ± 0.0 ^a^	0.12 ± 0.01 ^b^	0.23 ± 0.02 ^c^	n.d.
	Z-3-hexen-1-ol	0.05 ± 0.01 ^ab^	0.04 ± 0.01 ^ab^	0.03 ± 0.0 ^a^	0.05 ± 0.01 ^a^	0.20 ± 0.14 ^a^	0.06 ± 0.0 ^a^*	0.01 ± 0.01 ^a^	0.06 ± 0.0 ^b^	0.02 ± 0.0 ^a^	0.11 ± 0.01
**Hydrocarbons**	Dodecane	3.85 ± 0.58 ^a^	4.03 ± 0.11 ^a^	3.50 ± 0.55 ^a^	3.22 ± 0.15 ^a^	4.41 ± 0.59 ^a^	3.23 ± 0.74 ^a^	1.17 ± 0.11 ^a^	1.79 ± 0.59 ^a^	1.39 ± 0.08 ^a^	0.76 ± 0.02
	Tetradecane	1.13 ± 0.22 ^a^	1.19 ± 0.26 ^a^	0.71 ± 0.41^a^*	0.85 ± 0.01 ^a^	0.60 ± 0.07 ^b^	1.09 ± 0.10 ^c^	1.18 ± 0.06 ^a^	1.00 ± 0.06 ^a^	1.09 ± 0.15 ^a^	0.51 ± 0.01
	Tetradecene	1.22 ± 0.02 ^a^	1.17 ± 0.21 ^a^	1.09 ± 0.15 ^a^	1.32 ± 0.19 ^a^	1.18 ± 0.08 ^a^	1.02 ± 0.10 ^a^	2.66 ± 0.16 ^a^	2.49 ± 0.37 ^a^	2.31 ± 0.07 ^a^	8.62 ± 0.52
	2,4-heptadienal +pentadecan	174 ± 22.6 ^a^	208 ± 27.5 ^b^	160 ± 19.6 ^a^	146 ± 11.5 ^a^	164 ± 10.5 ^a^	172 ± 9.43 ^a^	36.0 ± 4.65 ^a^	51.4±8.07 ^a^	86.5 ± 0.52 ^b^	11.0 ± 0.11

^a–c^ Different subscript letters define statistically significant difference (*p* < 0.05) among different stages of the refining process for each oil; * Concentration of volatiles in deodorized oils marked with * do not differ statistically (*p* < 0.05) from control sample; Deodorized = refined; n.d.—not detected; *n* = 12.
